# Cholera in Boston, Mass.

**Published:** 1854-11

**Authors:** E. B. Moore

**Affiliations:** Boston, Mass.


### CHOLERA IN BOSTON.

BY E. B. MOORE, M. D., BOSTON, MASS.

(From a letter to one of the Editors.) * * We are now surrounded by a cholera atmosphere, and although the disease has not yet become an epidemic, yet many have fallen victims to its withering influence. Some physicians in this city and its immediate environs have been suddenlv removed by it, though in the case of some the fatal end was supposed to have been induced more by mental and moral causes, than even by previous exhausting labors and cases. Proper hygienic regulations, and even a very moderate attention to personal habits, is believed to be sufficient to protect one from the disease.

As your city may become afflicted with this disease, if it has not already been visited by it, and your services will be called into requisition to stay its progress, I have thought a few suggestions as to my mode of treatment, might not be wholly uninteresting to you. You are aware that I have had considerable experience in its treatment, and that my success has been fully equal to that of any others. During the epidemic of 1849, while you were with me, you remember I had the medical charge of one of the districts of this city, where the disease was more prevalent than in any other part of the city, and that I had an ample opportunity to try the effects of various remedies and modes of treatment, and to test their comparative efficacy; an opportunity, in fact, much more abundant and absolute, than usually falls to the lot of those physicians whose observations are restricted to the rounds of ordinary private practice, however extensive. Both during that epidemic and the present season, if called early, the disease was almost always arrested, and this circumstance, more than any other, constitutes the turning-point of the case.

The indications of cure are to allay the spasms, to stop the vomiting and purging, and to restore the secretions of bile and urine; these accomplished, the patient is well. To allay the spasms, and to put a stop to the vomiting, I give as follows:

℞ Sps. Lavend Comp.

Tinct. Opii Camph. *aa* ʒij

Muc. Acac. ʒiij

Chloroform ʒj

Aq. Cinnamonie ℥j M

Dose, a teaspoonful every half hour, or oftener if urgent, in a little cold water, and if vomited up, to be repeated again immediately. Apply mustard paste to the epigastrium, rub the extremities with *dry hot* flannels, and wrap the whole body in the same. Give no drinks, except perhaps occasionally a little ice water, or a piece of ice, if thirst is urgent.

This course will freqently fulfill the first three indications. If the purging is not stopped in one-half or three-fourths of an hour, I give a quarter of a grain of Sulph. Morphiæ, made in a pill with Pulv. Acacia, every two hours, but if the case is very urgent, somewhat more frequently. In addition to hot flannels, I order bottles of hot water to the feet and about the body. As soon as the cramps, vomiting and purging are stopped, which will generally be in the course of three hours, and sometimes much sooner, I endeavor to restore the secretions of bile and urine. For this purpose, to invite the bile, I generallv employ the following prescription, which though mild, rarely fails of accomplishing a favorable effect:

℞ Pulv. Opii, grs. iij
Hydr. c. creta " xij
Muc. Acacia 2 s.
M. Ft. pl. No. vj—one every two hours.

To restore the secretion of urine, I think by far the most appropriate means that can be employed, is Chlorate of Potass. which I give in five grain doses, every two hours, alternating with the pills above mentioned.

This then is the mode of treatment upon which I rely most, in my cenflicts with this fearful disease. I have not been guided in the choice of this treatment by any of the numerous fancies and theories respecting the intricate character of cholera. I have simply asked myself what are its obvious phenomena, and what mode of meeting these morbid tendencies, most readily suggests itself to the consideration, and commends itself to the judgment. My individual testimony is, that this course I have found more successful than any other plan of treatment, and this consideration amply compensates for any lack of startling novelties it may possess.

This year, thus far, I have not lost a single patient with the cholera, notwithstanding I have had many cases which at first view, appeared almost desperate.

As a remedy to check the premonitory diarrhœa of cholera, and also as an excellent remedy for chronic diarrhœa supervening from other causes, I have found nothing equal to the Liquor Ferri Pernitratis, given in doses of ten to twentv drops once in three or four hours.

BOSTON, Aug. 14, 1854.

